# Biomarkers of Response to Venetoclax Therapy in Acute Myeloid Leukemia

**DOI:** 10.3390/ijms25031421

**Published:** 2024-01-24

**Authors:** Carlos Rodríguez-Medina, Ruth Stuckey, Cristina Bilbao-Sieyro, María Teresa Gómez-Casares

**Affiliations:** 1 Hematology Department, Hospital Universitario de Gran Canaria Dr. Negrín, 35019 Las Palmas de Gran Canaria, Spain; crodmed@gobiernodecanarias.org (C.R.-M.); rstuckey@fciisc.es (R.S.); bilbaocristina@gmail.com (C.B.-S.); 2 Morphology Department, Universidad de Las Palmas de Gran Canaria, 35016 Las Palmas de Gran Canaria, Spain; 3 Department of Medical Sciences, Universidad de Las Palmas de Gran Canaria, 35016 Las Palmas de Gran Canaria, Spain

**Keywords:** acute leukemia, apoptosis, BCL2-family proteins, biomarkers, genetics, chemotherapy

## Abstract

Recent progress in the use of massive sequencing technologies has greatly enhanced our understanding of acute myeloid leukemia (AML) pathology. This knowledge has in turn driven the development of targeted therapies, such as venetoclax, a BCL-2 inhibitor approved for use in combination with azacitidine, decitabine, or low-dose cytarabine for the treatment of newly diagnosed adult patients with AML who are not eligible for intensive chemotherapy. However, a significant number of AML patients still face the challenge of disease relapse. In this review, we will explore biomarkers that may predict disease progression in patients receiving venetoclax-based therapy, considering both clinical factors and genetic changes. Despite the many advances, we conclude that the identification of molecular profiles for AML patients who will respond optimally to venetoclax therapy remains an unmet clinical need.

## 1. Introduction

Acute myeloid leukemia (AML), the second most frequent leukemia in adults, is characterized by both clinical and biological heterogeneity and poor prognosis. In recent years, significant progress has been made in our understanding of the molecular mechanisms of AML as a result of advances in genomic techniques, in particular next-generation sequencing (NGS). Indeed, the analysis of a number of genes whose mutations have clinical implications is essential for the accurate risk stratification of patients with AML according to international recommendations, such as those made by the European LeukemiaNet (ELN) [1,2,3]. Nevertheless, individual patients with AML show high variability in their individual mutation profiles [4].

AML was classically defined by the detection of an accumulation of >20% immature hematopoietic myeloid cells in the bone marrow, although the revised 2022 World Health Organization (WHO) classification and recently published International Consensus Classification (ICC) removed the 20% blast requirement for patients with defining genetic abnormalities, thus giving greater importance to a molecular-defined classification of the disease [5,6]. This reduction of the blast threshold has clouded the distinction between AML and myelodysplastic syndrome/acute myeloid leukemia (MDS/AML), with 10–19% blasts in blood and bone marrow. Indeed, 30–40% of MDS patients will progress to AML (and will be included in the AML entity with myelodysplasia-related changes (AML-MRC)). As such, understanding the molecular events that lead to AML transformation from MDS is essential, and has been reviewed by Bănescu and colleagues in this Special Issue [7].

Proliferative events and maturation arrest were initially postulated to predominate in leukemogenesis models [8]; however, NGS studies in AML have revealed that the apoptosis pathway is frequently affected. Indeed, the capability of tumor cells to avoid programmed cell death has long been considered one of the hallmarks of cancer, with an accumulation of supportive evidence since the 1970s [9].

Specifically, increasing evidence is revealing the importance of the BCL-2 protein family in leukemogenesis. Malignant blasts have the ability to evade apoptosis via the constitutively elevated expression of antiapoptotic proteins of the BCL-2 family. Molecular studies have confirmed these observations and demonstrated the prognostic impact of high levels of expression of *BCL2*, measured by immunohistochemistry [10] or mRNA quantification [11,12]. Moreover, high levels of BCL-2 family proteins are associated with resistance to chemotherapy [10,13,14].

Scientific interest in this biological mechanism in relation to leukemia has increased in recent years due to the discovery of specific agents targeted against BCL-2 that have demonstrated efficacy in chronic lymphocytic leukemia (CLL) and AML [15]. In clinical trials, Navitoclax (ABT-263) as a single agent significantly reduced tumor burden in most patients with CLL. However, dosing was limited due to thrombocytopenias [16,17]. However, a second BCL-2 inhibitor, ABT-199, was better tolerated than ABT-263 in clinical trials because it does not target Bcl-XL and thus did not reduce platelet survival [18,19]. In 2016, the United States Food and Drug Administration (FDA) and European Medicines Agency (EMA) approved the (first) targeted BCL-2 inhibitor, venetoclax (ABT-199, BH3 mimetic), for the treatment of adult patients with CLL (with 17p deletion or *TP53* mutation who are unsuitable for or have failed a B-cell receptor pathway inhibitor, or who have failed both chemoimmunotherapy and a B-cell receptor pathway inhibitor). 

The recent special issue of International Journal of Molecular Sciences on “Advanced Research in AML”, which includes three original articles and two reviews, provides new information on molecular mechanisms with a role in AML that can stimulate the development of novel targeted therapies for the treatment of patients with AML. For example, one featured study describes how *RUNX1* mutations in AML, which promote hypermethylation by attracting TET2 demethylase to their binding sites (TFBS), were associated with chemotherapy resistance [20]. The study also revealed that demethylation therapy restored expression of *BIK*, a downstream target of *RUNX1*, restoring sensitivity to chemotherapy as a result. 

Despite progress in the area of personalized medicine, approximately 85% to 89% of AML patients will relapse in less than three years [21,22]. Thus, there is a clear need for research into biomarkers that can identify AML patients who may benefit from targeted therapies or predict responses to novel treatments. The objective of this review is to examine key biomarkers for guiding decisions on the initiation of anti-BCL-2 therapy in AML patients, focusing on venetoclax.

## 2. Apoptosis Pathway in AML: An Overview

The cellular mechanisms of apoptosis and autophagy are regulated by two pathways: the extrinsic pathway, which responds to environmental stimuli such as tumor necrosis factor alpha or FASlig; and the intrinsic pathway. Intrinsic apoptosis is a form of regulated cell death that can be initiated by a variety of microenvironmental perturbations including but not limited to growth factor withdrawal, DNA damage, endoplasmic reticulum stress, overload of reactive oxygen species (ROS), replication stress, microtubular alterations, or mitotic defects [23,24]. Finally, any of these pathways can in turn lead to the activation of caspases that are ultimately responsible for the degradation of essential cellular proteins.

**Figure 1:**
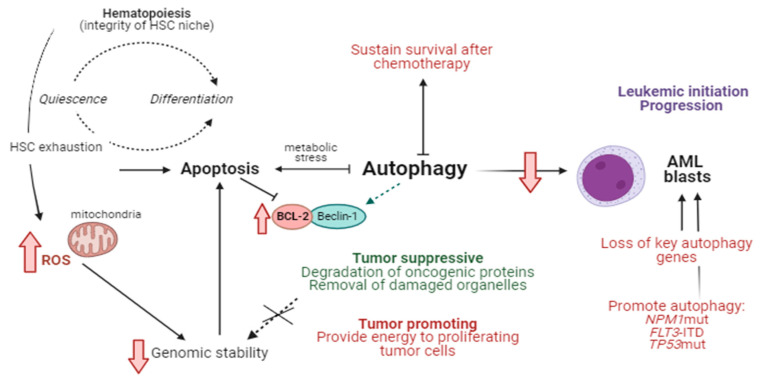
Autophagy allows cells to alter metabolism and thus adapt to stress. This adaptation is important to maintain the integrity of the hematopoietic stem cell (HSC) niche by balancing quiescence and differentiation. In cancer, autophagy has a protective (tumor suppression) function, via the degradation of oncogenic proteins and damaged organelles, such as mitochondria, that can produce high levels of reactive oxygen species (ROS), thus threatening genomic integrity. Indeed, inhibited autophagy can raise ROS levels and cause oxidative DNA damage and subsequently HSC exhaustion In addition, autophagy can play a malignant (tumor promoting) role by providing energy to proliferating tumor cells. In AML blast cells, autophagy is reduced, which limits nutrient availability, causing metabolic stress that can result in apoptosis. Moreover, the loss (or mutation) of key autophagy genes, such as *ATG5*, has been associated with leukemia initiation, and autophagy suppression can delay AML progression. Importantly, autophagy suppression prolongs survival after chemotherapy and can confer resistance of leukemic cells to cytotoxic drugs. The link between autophagy and apoptosis is mediated by Beclin-1, which is bound to and inhibited by BCL-2 or BCL-XL. In addition, mutations to factors such as *NPM1* and *FLT3*-ITD enhance autophagy, conferring a survival benefit to AML blasts [23,24,25,26,27,28]. Figure created with Biorender.com.

Autophagy plays a complex role in AML (for a recent review, see [25]) and can be inhibited by the binding of the BCL-2 protein [26]. On the one hand, autophagy can promote the survival and growth of AML cells by helping them adapt to stressful conditions and providing necessary nutrients (Figure 1). This pro-survival aspect of autophagy can contribute to AML cells’ resistance to chemotherapy [27]. For example, *NPM1*-mutated AML has been shown to have high autophagy activity that enhances the survival of leukemic cells. Indeed, a recent study published in this Special Issue revealed that the cytoplasmic localization of the tumor protein p53 inducible nuclear protein 2 (TP53INP2) in *NPM1*-mutated cells increased autophagy by promoting the interaction of microtubule-associated protein 1 light chain 3 (LC3) with autophagy-related 7 (ATG7), thus promoting leukemogenesis [28].

## 3. The Role of BCL-2 and the BCL-2 Superfamily in Apoptosis

There is a wide range of means to inhibit apoptosis. In the seminal paper by Hanahan and Weinberg, resistance to suppressor signals is highlighted as a fundamental characteristic of oncogenesis [9]. Typically, the intrinsic pathway is inhibited in cancer, although not responding to external stimuli for apoptosis is also considered important.

Regulation of the intrinsic pathway of apoptosis is governed by the BCL-2 superfamily of proteins. The BCL-2 family proteins can be classified based on their structure and Bcl-2 homology (BH) domains. The anti-apoptotic members BCL-2, BCL-XL, BCL-W, MCL-1, and BFL-1/A1 possess four BH domains, BH1-BH4, and present a hydrophobic groove in their structure that binds to the BH3 domain found in the pro-apoptotic proteins. The pro-apoptotic effectors, BAX, BAK, and BOK, possess three to four BH domains, and have the capacity to form pores in the mitochondrial outer membrane. These domains are composed of nine α-helices, including a transmembrane C-terminal α-helix that can anchor to the mitochondrial outer membrane. Their tridimensional structures are very similar to the membrane pore-forming domains found in diphtheria toxin and colicins (Table 1) [29].

Under normal conditions, the integral outer mitochondrial membrane protein BCL-2 and its family members (MCL-1, BCL-XL, BCL-W, and BFL-1/A1) constrain the BAX and BAK mediators of mitochondrial outer membrane permeabilization. Pro-apoptotic signals activate BID and BIM, which are usually bound to and sequester the effector proteins (BAX/BAK). Upon stress, the sensitizers, such as BAD, bind to and inhibit the anti-apoptotic suppressors. This binding leads to the release of the effector proteins and their activating dimerization, resulting in mitochondrial outer membrane permeabilization, which is the point of no return in the intrinsic pathway of apoptosis (reviewed in [24,31]).

Following activation of the intrinsic pathway, a complex interplay between various members of the BCL-2 family of proteins culminates in the release of cytochrome-C from “stressed” cells’ mitochondria by the creation of pores in the mitochondrial outer membrane, a process denoted as mitochondrial outer membrane permeabilization. Extra-mitochondrial cytochrome-C complexes with APAF-1, which subsequently oligomerizes into a heptamer known as the apoptosome and recruits and activates caspase 9. Activated caspase 9 plays a central role in cleaving other effector caspases, and this ultimately leads to the proteolytic dismantling of the cell.

## 4. Role of Apoptosis in Leukemogenesis

The process of leukemogenesis, or the development of leukemia, has been extensively investigated in relation to apoptosis mechanisms. While initially it was believed that the main drivers of leukemia were cell proliferation and the blocking of cell maturation [8], recent research, supported by high-throughput sequencing techniques, has revealed a more complex landscape. It has been discovered that over 40 genes and up to 11 molecular pathways interact to trigger leukemogenesis [1]. In this process, resistance to programmed cell death, or apoptosis, plays a prominent role, with an emphasis on mutations affecting genes such as *TP53*, *PHF6*, and *WT1*, according to studies such as those conducted by the Cancer Genome Atlas Research or the consortium led by the Wellcome Trust Sanger Institute, Ulm University, and the European Bioinformatics Institute/European Molecular Biology Laboratory [1,4].

Recent studies have highlighted the crucial role that TP53 plays in the model of leukemogenesis. *TP53* inactivation induced by gene mutation or deletion favors the activities of oncogenes, thereby promoting uncontrolled proliferation of cancer cells [32]. Indeed, mutations or chromosomal alterations affecting the *TP53* locus are present in up to 10% of AML cases [33]. Also, mutant *TP53* is strongly associated with large structural and complex chromosomal aberrations, as illustrated by its co-occurrence with a complex karyotype, which is associated with reduced overall survival (OS) in myeloid malignancies [33]. The impact of *TP53* mutations is so significant that authors such as Grimwade and Papaemmanuil consider that mutations in this gene can be regarded as a driver in the leukemogenesis process in a subgroup of AML patients [1,34]. Consequently, it is classified as a distinct entity within AML [1,34], recognized by the ICC but not the WHO [5,6].

Since TP53’s function is to inhibit cell division and proliferation in situations of genotoxicity, it can induce the intrinsic apoptosis pathway by directly activating the effectors BAX/BAK [23]. The sum of the effects of TP53 activation are pro-apoptotic and, in part through its interactions with the BCL-2 superfamily, TP53-deficient cells are rendered chemotherapy resistant [24].

For more than two decades, the impact of high levels of *BCL2* on AML has been studied. Overexpression of the BCL-2 protein in AML tissues has been demonstrated using flow cytometry techniques and was associated with increased chemotherapy resistance [10]. Similar findings have been replicated using PCR techniques to quantify *BCL2* mRNA [11,35]. More recently, our group found that the persistence of high levels of *BCL2* mRNA after induction was associated with a poor prognosis [14]. These findings were confirmed for other BCL-2 family proteins such as BIK and *BCL2L13* [36].

Similarly, the proapoptotic gene *WT1* has been extensively studied. Mutations in *WT1,* as well as its overexpression, have been well documented in AML, with mutations detected in 12 of the 200 AML patients (6%) studied by NGS [4]. High expression of *WT1* has been associated with poor prognosis at the time of diagnosis [37], and some authors have postulated its persistence as a possible follow-up marker [38,39]. The expression of other genes may also have prognostic value. In fact, several multi-gene scores have been developed to predict which AML patients, achieving complete remission (CR) in response to standard induction therapy, will have longer survival [12,40,41]. The application of these scores may help distinguish patients who will require a cell therapy-based approach from those who are chemosensitive and thus might avoid allogeneic hematopoietic stem cell transplantation (HSCT). 

In contrast to AML, there is less evidence regarding the impact of overexpression of apoptosis-related proteins in acute lymphoblastic leukemia (ALL), especially BCL-2, where expression levels exhibit higher variability. Nevertheless, in pediatric ALL cell lines, BCL-2, BCL-XL, and BAX expression has been found to be TP53-dependent and correlated with apoptosis sensitivity, suggesting a role for these proteins in the regulation of apoptosis in ALL [42]. 

In conclusion, the available evidence suggests that proteins involved in the intrinsic apoptosis pathway, particularly those in the BCL-2 superfamily, play a crucial role in the development of both AML and ALL, justifying their use as therapeutic targets [43].

## 5. Antiapoptotic Agents in AML

Based on this theoretical background, various strategies have been sought to induce apoptosis via targeted therapy, particularly by sequestering the function of the BCL-2 family of proteins. Since apoptosis is impaired in malignant cells that overexpress pro-survival BCL-2 proteins, drugs mimicking their natural antagonists, BH3-only proteins, might overcome chemoresistance [44].

The first pharmacological approach tested in patients with acute leukemias was the anti-sense oligonucleotide Oblimersen sodium (Genasense), which targets the first six codons (the initiation codon region) of *BCL2* mRNA. By doing so, it inhibits *BCL2* mRNA translation and reduces BCL-2 protein levels [45]. In preliminary clinical trials in acute leukemias, when combined with chemotherapy, it demonstrated biological activity by significantly reducing *BCL2* mRNA expression levels. However, in a phase 3 randomized controlled trial (RCT) as a first-line treatment combined with chemotherapy, there was no clinical benefit observed. As a result, its clinical development for acute leukemias came to a halt [43]. Interestingly, this drug was not tested in elderly AML patients in combination with hypomethylating agents (HMA).

The first BH3 mimetic developed in the field of AML was obatoclax, a small molecule that acts as a pan-inhibitor of BCL-2 family proteins, with pro-apoptotic activity. Obatoclax, also known as GX015-070, selectively antagonizes the BH3-binding groove of the BCL-2 family. This molecule has the capacity to inhibit not only BCL-2 but also other members of the BCL-2 family, such as BCL-X, MCL-1, BCL-B, and BCL-W, acting as a pan-inhibitor [46]. However, clinical results with obatoclax have been modest, leading to its abandonment in favor of more potent and selective molecules, such as ABT-737.In vitro, ABT-737 has demonstrated its ability to induce apoptosis by disrupting the heterodimerization of the BCL-2/BAX complex [47]. Nevertheless, studies in animal models showed that ABT-737 required synergistic action with drugs that downregulated MCL-1 [48].

In the case of the oral BH3 mimetic ABT-263 (navitoclax), which has a high binding affinity for BCL-2, BCL-XL, and BCL-W, it was shown to disrupt protein–protein interactions between BCL-2/BCL-XL and the effector proteins, such as BAX. Despite promising in vitro results, its development in AML has been put on hold due to limiting toxicity related to platelet loss mediated by BCL-X1 [43]. Indeed, a recent drug screening study demonstrated that AML with erythroid or megakaryocytic differentiation, such as M7, depend on BCL-XL rather than BCL-2, showing higher sensitivity to navitoclax and reduced sensitivity to venetoclax [49].

Platelets play a crucial role in protecting malignant cells against chemotherapy-induced apoptosis [50]. Additionally, AML patients with thrombocytopenia have an increased risk of bleeding. An original article published in this Special Issue identified three biomarkers (*CSF1R*, *TNFSF15*, and *CLEC10A*) whose expression was associated with reduced platelet levels in normal karyotype AML. These biomarkers could potentially help predict which AML patients are at an increased risk of thrombocytopenia [51].

Following studies with navitoclax and crystallography techniques, researchers were able to examine the binding pocket of navitoclax to BCL-X and BCL-2. This led to the design of an inhibitor with greater specificity for BCL-2, avoiding the hematological toxicities associated with binding to BCL-X. This marked the development of ABT-199 or venetoclax. Venetoclax quickly demonstrated its effectiveness in three patients with refractory CLL, where responses were observed in the form of tumor lysis syndrome [52]. Venetoclax has a sub-nanomolar affinity for BCL-2, making it a potent activator of apoptosis. It allows for the dimerization of BAX-BAK by binding to the BH3 domain of BCL-2 and preventing it from sequestering BH3 activating proteins like BIM and BID.

However, it is important to consider the choice of AML cell line for in vitro research into treatment response, as highlighted in this Special Issue [53]. This is because different cell lines harbor distinct genetic alterations, some of which are associated with specific AML subtypes. For example, the SKNO-1 cell line contains the *RUNX1::RUNX1T1* fusion gene, monosomy 17, and *TP53* mutation and overexpression. These alterations could dramatically affect the cell line’s response to venetoclax or other BCL-2 family inhibitors.

## 6. Venetoclax Therapy in Acute Leukemia

The clinical development of venetoclax in acute leukemia has been rapid. In a phase 2 trial carried out at the MD Anderson Cancer Center (MDACC), researchers demonstrated modest clinical activity in the form of a 19% CR rate and a 38% overall response rate (ORR) with venetoclax monotherapy in a cohort of relapsed/refractory (R/R) AML patients, with up to 40% having received three or more prior lines of treatment [54]. This led to the development of clinical trials combining venetoclax with HMA and low-dose cytarabine (LDAC) in first-line therapy for AML patients who were not candidates for intensive chemotherapy. This approach was based on the hypothesis, supported by biological models, that a synergistic effect existed between HMA and anti-BCL-2 therapy, particularly due to HMA’s ability to inhibit one of the mechanisms of resistance to anti-BCL-2 therapy, the overexpression of MCL-1 [55].

The initial phase 1b/2 trial of venetoclax enrolled 147 AML patients, initially exploring doses of 400, 800, and 1200 mg, with the first two doses selected for the phase 2 part of the trial. Response rates did not differ significantly between doses, but substantial clinical activity was confirmed. It showed CR or CR with incomplete hematologic recovery (CRi) rates of 68%, achieved rapidly within 1.2 months. This translated to a median survival of 15 months, which was an advantage compared to historical controls treated with HMA who had a median survival of around 10 months [15,56]. These results were corroborated by the phase 3 VIALE-A clinical trial [57], leading to regulatory approval of the combination therapy of venetoclax with azacitidine (AZA) or decitabine or LDAC by the FDA on 21 November 2018, for the treatment of newly diagnosed AML in adults aged 75 or older, or for those who have comorbidities that would prevent an intensive induction regime. Since its approval, venetoclax has become a new standard of care for AML patients who are not candidates for intensive chemotherapy. Meanwhile, multiple combinations of these drugs with new agents or classical chemotherapy are being developed to expand their indications.

### BCL-2 Family Inhibition in ALL

An initial preclinical study demonstrated the sensitivity of ALL cells to venetoclax [58]. Interestingly in ALL, BCL-XL expression is a key predictor of a poor response to venetoclax in both T-ALL cell lines and T-ALL patient samples. This study was also important because it suggested that the differentiation stage of the T-ALL clone influences its reliance on specific apoptosis-related proteins. For example, early T-cell progenitor (ETP) T-ALL is dependent on BCL-2, while other T-ALL types depend on BCL-XL.

Venetoclax is currently being tested in numerous clinical trials for both newly diagnosed and R/R ALL. Combining venetoclax with the BCL-XL inhibitor navitoclax has shown positive response rates in R/R ALL patients [59]. Notably, *KMT2A*-rearranged ALL xenografts responded well to venetoclax as monotherapy, both in vitro and in vivo [60]. A recent review published in the International Journal of Molecular Science summarizes the venetoclax-based therapy regimes undergoing development in various combinations in ALL [61], especially in ETP T-ALL.

## 7. Biomarkers to Predict Response to Anti-Apoptotic Therapy in AML

There are currently no predictive laboratory markers of relapse and resistance to venetoclax treatment. Many patients experience severe side effects (grades 3–4) such as neutropenia and anemia, with more than 70% of patients enrolled in the VIALE A randomized trial requiring a dose modification of venetoclax because of hematological toxicities of grade 3 and above [57]. Other common side effects include pneumonia, upper respiratory tract infections, nausea, diarrhea, fever, and fatigue. Thus, given that the medication is indicated for patients who are generally of advanced age and/or have comorbidities, the identification at baseline of patients likely to respond well to venetoclax treatment, and the early discrimination of patients who would benefit from an additional or alternative therapy, is an innovative and cost-effective way to improve the level of health and quality of life of patients.

At first sight, one might think that simply measuring *BCL2* levels at diagnosis, either by immunohistochemistry or mRNA quantification, could predict the response to anti-BCL-2 treatment, since higher BCL-2 protein levels correlate with a higher sensitivity to venetoclax [62]. In fact, it seems that *BCL2* levels alone do not provide a good explanation for this sensitivity, but rather, it is the amount of BCL-2 capable of binding and sequestering proapoptotic proteins like BIM and BAX that truly matters [63,64,65].

### 7.1. Karyotype

It is important to emphasize that the studies in elderly patients that supported the commercial approval of venetoclax in combination with HMA were in individuals not eligible for intensive chemotherapy and in which favorable cytogenetics were underrepresented. However, more recent research into venetoclax in combination with intensive chemotherapy in the frontline setting has enabled the study of the drug’s effects on patients in all three genetic risk groups, as defined by the 2022 ELN criteria [3], thus some information about the impact of the karyotype can be obtained.

Firstly, in the context of patients not eligible for intensive chemotherapy, the results of the VIALE-A and VIALE-C studies showed a higher probability of achieving a composite CR (including CR and CR with incomplete hematologic recovery) for both the intermediate-risk group (74.2% vs. 31.5%) and the adverse-risk group (52.9% vs. 23.2%) comparing venetoclax plus AZA vs. AZA plus placebo, respectively [57,66].

An interesting observation is that the composite CR for the subgroup with *TP53* mutations was 40.8% vs. 16.7% in a specific pooled analysis of patient data from the VIALE-A and PHIb trials, comparing AZA + venetoclax vs. AZA + placebo, respectively [57].

However, while remission rates were improved for higher cytogenetic risk subgroups, the OS of patients with both a *TP53* mutation and poor-risk cytogenetics was not improved by the combination treatment (Table 2), although the OS of *TP53*^WT^ patients with poor risk cytogenetics was improved by AZA + venetoclax, albeit with a modest impact.

Importantly, single- and multi-hit *TP53* mutation was found to be frequent in AML patients who did not respond to venetoclax therapy in combination with HMA or low-dose AZA from several studies [75]. Therefore, together with complex karyotype, these mutations are recognized as the most common cause of primary resistance to venetoclax combination therapy [76].

Another recent study reported real-world experience with elderly patients and favorable molecular genetic characteristics, albeit with a median patient age of 70 years and a 44% relapse rate. In this cohort, a CR/CRi rate of 80% was reported in 10 patients with core-binding factor (CBF) AML using the combination of venetoclax with HMA [77]. In a second study of 30 CBF-AML patients with a median age of 40 years, and medical conditions that made them ineligible for intensive chemotherapy (generally active infections), treated with one cycle of venetoclax and HMA, 70% achieved CR/CRi [78]. More interestingly, it was observed that in the 13 patients with t(8;21), the CR/CRi rate with one cycle was 30%, and for the 17 patients with inv(16), 100% achieved CR/CRi but with suboptimal levels of molecular response.

Overall, even though NGS studies in CBF-AML have revealed that the mutational profile is dominated by events in cellular proliferation pathways like RAS, *KIT*, or *FLT3*, these findings suggest that the use of anti-apoptotic agents might be of particular interest in this patient group. Clinical trials are underway to test this hypothesis, especially in younger patients (i.e., the NCT04628026, AMLSG, and HOVON trials).

Ongoing studies involving younger AML patients show interesting results in both CR and OS [79,80]. However, considering that this population has a high incidence of allogeneic transplant after the first CR, it is worth assessing the impact of adding venetoclax to these patients. Ongoing clinical trials will explore whether the combination of intensive chemotherapy with venetoclax is indeed useful, especially by investigating whether it effectively improves the quality of CR with an increase in MRD negativity.

For the favorable cytogenetic risk group, studies conducted testing venetoclax in combination with CLIA (cladribine, idarubicin, and cytarabine) or FLAG-Ida (fludarabine, cytarabine, granulocyte colony-stimulating factor, and idarubicin) have demonstrated CR rates exceeding 90% [68]. Furthermore, and perhaps even more importantly, they achieved measurable residual disease (MRD) clearance. Similar positive results have been published for patients under 60 years of age who received the combination of 7 + 3 with venetoclax in the phase 2 ChiCTR2000041509 study [67], and more recently by the Italian GIMEMA 1718 trial (NCT03455504), which evaluated the combination of venetoclax with FLAI induction (fludarabine, idarubicin, and cytarabine) [81]. More recent results from the latter study presented at the 2023 annual congress of the European Hematology Association reported an increase in CR and MRD negativity (level < 0.1%) of 84% and 74% for patients with a non-favorable ELN risk who received the venetoclax combination vs. 63% and 40% for standard induction, respectively [69]. Therefore, the addition of venetoclax to intensive chemotherapy combinations for young patients could play an important role in the depth of responses achieved, although the results published to date have not translated into an impact on OS.

### 7.2. Mutations

The mutational background plays a crucial role in the response to venetoclax, as first demonstrated in patients with CLL treated with venetoclax. The development of mutations in *BCL2*, such as p.G101V, which results in reduced binding to venetoclax, has been associated with disease progression while on venetoclax [82]. In CLL, mutations in the apoptosis effector *BAX* were identified in 32% of patients (13 out of 41) [83]. Other studies on CLL patients revealed that those with somatic mutations in *SF3B1*, *NOTCH1*, or chromosome 17p deletion had a shorter duration of response to venetoclax monotherapy [84]. In addition, progression on venetoclax was associated with the development of *CDKN2A/B* deletions and *BTG1* missense mutations, which may confer a clonal advantage [85]. The acquisition of an 8p deletion or 1q gain, the latter causing overexpression of *MCL1*, also contributed to disease progression [86].

In the case of AML patients, as previously mentioned, *TP53* mutations were associated with the lowest survival on venetoclax with HMA. On the other hand, there are some molecular profiles that are associated with a higher likelihood of responding to venetoclax therapy.

For instance, particularly promising CR rates and high relapse-free survival rates were observed in *NPM1*-mutated AML patients who received venetoclax with HMA or LDAC in pivotal trials (Table 2) [75]. However, the mechanism by which *NPM1* mutation leads to venetoclax sensitivity remains to be elucidated. For example, in the pivotal VIALE-A clinical trial, the CR rate for the *NPM1*-mutated group was 66.7% compared to 23.5% in the control group, with a trend toward improved OS [56]. This trend reached statistical significance in a subsequent analysis by Pollyea et al. when combining patients from various venetoclax development clinical trials [70].

Similarly, promising clinical results of the AZA + venetoclax combination for the treatment of MRD positivity in young patients with *NPM1*-mutated AML have been published [87], while the results of the GIMEMA group’s clinical trial testing this hypothesis are pending publication [88]. All this evidence supports the hypothesis that *NPM1* mutation status may be one of the most sensitive predictive biomarkers of response to venetoclax.

Likewise, patients with mutations in *IDH1/IDH2* had favorable outcomes to venetoclax combination therapies, confirming results from preclinical models where *IDH1/IDH2* mutation increased BCL-2 dependence, resulting in higher venetoclax sensitivity [89,90,91]. In both the VIALE-A and VIALE-C clinical trials, significantly higher rates of CR were observed for these subgroups (Table 2), translating into a statistically significant clinical benefit with median survival times in patients not eligible for intensive chemotherapy exceeding 20 months for the *IDH2* subgroup. Moreover, venetoclax combination treatment regimes improved survival for both *IDH1*^mut^/*NPM1*^mut^ and *IDH2*^mut^/*NPM1*^mut^ vs. *IDH1*^mut^*/NPM1*^WT^ and *IDH2*^mut^/*NPM1*^WT^, respectively [92].

The mechanism of increased sensitivity is caused by an inhibition of mitochondrial respiration and mitochondrial cytochrome c oxidase by the metabolite 2 hydroxyglutarate, which lowers the apoptosis induction threshold [89,93]. As a result, triplet studies involving the IDH1 and IDH2 inhibitors, ivosidenib (NCT03471260) and enasidenib (NCT04092179), in combination with venetoclax + AZA are underway.

ASXL1 acts as an epigenetic regulator via PRC2-mediated chromatin modification, maintaining various genes in a repressed state. Recently, *ASXL1* has been included by the WHO and the ICC as one of the genes whose mutation defines AML related to myelodysplasia. *ASXL1* mutation resulted in higher chromatin accessibility on the *BCL2* locus, leading to *BCL2* overexpression [94]. In a retrospective study of 40 patients, *ASXL1* mutations predicted a favorable response and survival after venetoclax and HMA therapy in patients with myelodysplastic syndromes (MDS) with excess blasts (EB), with rates of complete remission of 92% for patients with *ASXL1* mutation vs. 57% for those without [74].

Patients with spliceosome mutations in genes such as *SF3B1, SRSF2,* and *U2AF1*, treated with HMA + venetoclax, had similar composite CR to wild-type patients (79% vs. 75%, respectively) [95]. In this cohort of 119 patients with a median age of 72 years, including 39 with a spliceosome mutation, 34% were de novo, 40% secondary AML, and 24% treatment-related AML. Similarly, a second study of 994 de novo AML patients with a median age of 67 years, including 266 with a spliceosome mutation, confirmed similar response rates and survival outcomes for younger AML patients with a spliceosome mutation compared to wild-type, while for older AML patients with a spliceosome mutation, venetoclax reduced the risk of relapse and death [96].

More recently, favorable information has emerged regarding the likelihood of response in patients with a *DDX41* mutation. There is increasing evidence that germline mutations in this gene lead to the onset of myeloid neoplasms from the sixth to seventh decade of life. Authors from the MDACC reviewed a cohort of 3795 patients, identifying 151 individuals carrying a *DDX41* mutation [72]. They observed a particularly favorable prognosis for those treated with low-intensity regimens combined with venetoclax (Table 2). Similarly, Nanaa et al. retrospectively examined their series, identifying 12 patients with AML and MDS carrying the *DDX41* mutation [73]. The patients were treated with HMA plus venetoclax regimens, confirming a high rate of hematologic responses and a trend toward improved OS.

It is important to emphasize that the findings presented related to *ASXL1* and *DDX41* are based on single-center observations and the studies were retrospective in nature. Larger prospective studies are needed to confirm these findings before *ASXL1* and *DDX41* can be considered alongside *IDH1/2* and *NPM1* as therapy-selective biomarkers for venetoclax.

### 7.3. Other Molecular Mechanisms of Resistance to Venetoclax

Besides somatic mutations, AML patients can develop other genetic mechanisms of resistance to venetoclax. The classic mechanism of resistance to venetoclax is the overexpression of other anti-apoptotic BCL-2 family proteins, in particular MCL-1, which is itself not affected by venetoclax, via the amplification of chromosome 1. Similarly, the overexpression of BCL-XL, which can also bind to BIM and thus sustain the inhibition of mitochondrial apoptosis, can contribute to venetoclax resistance.

A novel genetic mechanism of resistance to venetoclax for an AML patient was presented at the 2023 EHA meeting [97]. A patient with mutated *TP53* exhibited clonal evolution during venetoclax treatment due to *BCL2* amplification. The patient’s *BCL2* expression increased from 6% of nuclei prior to venetoclax start, to 17% of nuclei (as determined by FISH analysis) with gain of monosomy 12 after 45 days of venetoclax with decitabine. The mechanisms of venetoclax resistance will not be discussed further here, but we recommend a recent review [98].

Apart from the genetic or molecular effects on venetoclax response, there is an indication that venetoclax may have other mechanisms of action, including an immune-mediated mechanism. In one study, venetoclax was found to activate T-cells by increasing the generation of ROS, enhancing their cytotoxicity against AML cells [99]. Consequently, various clinical trials are ongoing with venetoclax in combination with AZA and an immune checkpoint inhibitor, such as evorpacept (ASPEN-05) for CD47-SIPRa, pembrolizumab (BLAST MRD AML-2) for PD-1, and sabatolimab (STIMULUS-AML1) for TIM3.

Dynamic BH3 profiling (DBP) is a functional tool employed for drug-induced mitochondrial apoptotic priming as assessed by mitochondrial outer membrane permeabilization. The balance between anti-apoptotic and pro-apoptotic BCL-2 family members, determined by BH3 profiling, influences a cell’s priming status, establishing a correlation between the apoptotic threshold and tolerance for apoptotic stimuli. The use of this technique has revealed the capacity of BH3 mimetics like venetoclax to sensitize cancer cells to apoptosis; it has helped predict responses to venetoclax in patients with AML via baseline BH3 profiling and has also revealed potential resistance mechanisms [100]. Indeed, due to its successful readout of a cell’s proximity to the apoptotic threshold, DBP has been included as a prognostic marker in several clinical trials for AML, CLL, and ALL.

## 8. Conclusions

The continued advancement of sequencing techniques continues to elucidate the mechanisms of AML biology, holding the potential to guide targeted therapeutic approaches that could enhance AML clinical outcomes. The approach adopted by the WHO and the ICC in 2022, both emphasizing the definition of AML based on genetic markers, reflects the growing recognition of the genetic foundations of this complex disease. Nonetheless, while significant strides have been made in understanding AML genetics, we are still in the early stages of identifying the molecular profiles of patients who may respond optimally to emerging targeted therapies like venetoclax [101].

Unlike other drugs such as ivosidenib, enasidenib, or FLT3 inhibitors, which have been FDA-approved along with specific diagnostic tests, there is currently no single test that can determine who will respond to venetoclax. While venetoclax response is likely to differ according to AML subtype and other patient-specific factors, such as immune response, data from clinical trials and retrospective studies suggest that *NPM1, IDH1/2,* and perhaps *ASXL1* and *DDX41* could be response biomarkers, but further research is needed to confirm these findings.

The search for biomarkers that can help in identifying patients who are unlikely to respond well to standard induction regimens and thus would be suitable candidates for novel targeted therapies remains an area of unmet clinical need.

## Figures and Tables

**Table 1:** Summary of the roles of the key BCL-2 family proteins in the apoptotic pathway.

Proteins	Role
BCL-2, BCL-XL, BCL-W, MCL-1, BFL1-/A1	Anti-apoptotic multidomain BCL-2 family proteins. Regulate cell death by inhibiting BH3-only activators and the pore-forming ‘effector’ proteins by sequestration.
BID, BIM, PUMA	BH3-only pro-apoptotic ‘activators’. Bind to and inhibit the ‘effector’ proteins by sequestration.
BAD, NOXA	BH3-only pro-apoptotic ‘sensitizers’. Bind to and inhibit the anti-apoptotic proteins by sequestration.
BAX, BAK, BOK	Multidomain pro-apoptotic ‘effectors’. Form a pore to permeabilize the outer membrane of mitochondria, releasing cytochrome c (and other apoptogenic factors, e.g., SMAC/DIABLO) into the cytosol to assemble with APAF-1 and pro-caspase 9 to form the apoptosome ‘wheel of death’ [30].
APAF-1	Pro-apoptotic cytoplasmic protein. Release from BCL-XL sequestration by cytochrome c activation required for caspase activation.

**Table 2:** Complete remission and overall survival rates (if evaluated) for molecular subgroups of AML patients who received venetoclax treatment combinations. The comparisons are for venetoclax + treatment vs. treatment alone.

Molecular Subgroup	Treatment Comparison	Composite Complete Remission Rate	Overall Survival (Months)	Study Description	References
Adverse karyotype	HMA	53% vs. 23%	7.6 vs. 6	Subgroup analysis	[57]
	LDAC	32% vs. 10%		Subgroup analysis	[66]
	7 + 3	75%		Phase 2 clinical trial. No comparative group	[67]
	CLIA	95%		Phase 2 clinical trial. No comparative group	[68]
	FLAI	84% vs. 40% (historical control 7 + 3)		Phase 2 clinical trial. Propensity score weighting analysis with historical 7 + 3 control match-up	[69]
Intermediate karyotype	HMA	74.2% vs. 31.5%		Subgroup analysis	[57]
	LDAC	53% vs. 19%		Subgroup analysis	[66]
	7 + 3	83%		Phase 2 clinical trial. No comparative group	[67]
	CLIA	96%		Phase 2 clinical trial. No comparative group	[68]
	FLAI	84% vs. 40%		Phase 2 clinical trial. propensity score weighting analysis with historical 7 + 3 control match-up	[69]
*TP53-*mutated	HMA	55.3% vs. 0%		Subgroup analysis	[57]
*IDH1-*mutated	HMA	66.7% vs. 9.1%	15.5 vs. 2.2	Data pooled from Phase III and Phase Ib studies	[70]
	LDAC	57% vs. 33.%	10.8 vs. 9	Subgroup analysis	[57]
*IDH2-*mutated	HMA	86% vs. 11.1%	NR vs. 12.2	Data pooled from Phase III and Phase Ib studies	[70]
	LDAC	57% vs. 33%		Subgroup analysis	[57]
*NPM1-*mutated	HMA	66.7% vs. 23.5%	NR vs. 4.8	Retrospective analysis	[57,71]
	LDAC	78% vs. 57%	NR vs. 9.8	Subgroup analysis	[66]
*DDX41-*mutated	HMA	87%	91 vs. 60% at 2 years	Retrospective unicentral study Heterogeneity of treatments	[72]
	HMA	88%	29	Retrospective unicentral study. Low number of patients (n = 12) and included both LMA and MDS patients	[73]
*ASXL1-*mutated	HMA	92 vs. 57%		Retrospective unicentral study in MDS with excessive blasts	[74]

CLIA: cladribine, idarubicin, and cytarabine; FLAI: fludarabine, idarubicin, and cytarabine; HMA: hypomethylating agents; LDAC: low-dose cytarabine; MDS: myelodysplastic syndrome; NR: not reached.

## Data Availability

Not applicable.

## References

[1] Papaemmanuil E., Gerstung M., Bullinger L., Gaidzik V.I., Paschka P., Roberts N.D., Potter N.E., Heuser M., Thol F., Bolli N. (2016). Genomic classification and prognosis in acute myeloid leukemia. N. Engl. J. Med..

[2] Gargallo P., Molero M., Bilbao C., Stuckey R., Carrillo-Cruz E., Hermosín L., Pérez-López O., Jiménez-Velasco A., Soria E., Lázaro M. (2022). Next-Generation DNA Sequencing-Based Gene Panel for Diagnosis and Genetic Risk Stratification in Onco-Hematology. Cancers.

[3] Döhner H., Wei A.H., Appelbaum F.R., Craddock C., DiNardo C.D., Dombret H., Ebert B.L., Fenaux P., Godley L.A., Hasserjian R.P. (2022). Diagnosis and management of AML in adults: 2022 recommendations from an international expert panel on behalf of the ELN. Blood.

[4] Ley T.J., Miller C., Ding L., Raphael B.J., Mungall A.J., Robertson A., Hoadley K., Triche T.J., Laird P.W., Cancer Genome Atlas Research Network (2013). Genomic and epigenomic landscapes of adult de novo acute myeloid leukemia. N. Engl. J. Med..

[5] Khoury J.D., Solary E., Abla O., Akkari Y., Alaggio R., Apperley J.F., Bejar R., Berti E., Busque L., Chan J.K.C. (2022). The 5th edition of the World Health Organization Classification of Haematolymphoid Tumours: Myeloid and Histiocytic/Dendritic Neoplasms. Leukemia.

[6] Arber D.A., Orazi A., Hasserjian R.P., Borowitz M.J., Calvo K.R., Kvasnicka H.M., Wang S.A., Bagg A., Barbui T., Branford S. (2022). International Consensus Classification of Myeloid Neoplasms and Acute Leukemias: Integrating morphologic, clinical, and genomic data. Blood.

[7] Bănescu C., Tripon F., Muntean C. (2023). The Genetic Landscape of Myelodysplastic Neoplasm Progression to Acute Myeloid Leukemia. Int. J. Mol. Sci..

[8] Gilliland D.G., Griffin J.D. (2002). The roles of FLT3 in hematopoiesis and leukemia. Blood.

[9] Hanahan D., Weinberg R.A. (2000). The hallmarks of cancer. Cell.

[10] Campos L., Rouault J.P., Sabido O., Oriol P., Roubi N., Vasselon C., Archimbaud E., Magaud J.P., Guyotat D. (1993). High expression of bcl-2 protein in acute myeloid leukemia cells is associated with poor response to chemotherapy. Blood.

[11] Karakas T., Maurer U., Weidmann E., Miething C.C., Hoelzer D., Bergmann L. (1998). High expression of bcl-2 mRNA as a determinant of poor prognosis in acute myeloid leukemia. Ann. Oncol..

[12] Sánchez-Sosa S., Rodríguez-Medina C., Stuckey R., Florido Y., Santana G., González Martín J.M., Cruz-Cruz N., Torres-Ochando M., Fernández R., Sánchez-Farías N. (2022). Can the Gene Expression Profile of Patients with Acute Myeloid Leukemia Predict Complete Remission Following Induction Therapy?. J. Cancer.

[13] Russell N.H., Hunter A.E., Bradbury D., Zhu Y.M., Keith F. (1995). Biological features of leukaemic cells associated with autonomous growth and reduced survival in acute myeloblastic leukaemia. Leuk. Lymphoma.

[14] Bilbao-Sieyro C., Rodríguez-Medina C., Florido Y., Stuckey R., Sáez M.N., Sánchez-Sosa S., González Martín J.M., Santana G., González-Pérez E., Cruz-Cruz N. (2020). BCL2 Expression at Post-Induction and Complete Remission Impact Outcome in Acute Myeloid Leukemia. Diagnostics.

[15] DiNardo C.D., Pratz K., Pullarkat V., Jonas B.A., Arellano M., Becker P.S., Frankfurt O., Konopleva M., Wei A.H., Kantarjian H.M. (2019). Venetoclax combined with decitabine or azacitidine in treatment-naive, elderly patients with acute myeloid leukemia. Blood.

[16] Wilson W.H., O’Connor O.A., Czuczman M.S., LaCasce A.S., Gerecitano J.F., Leonard J.P., Tulpule A., Dunleavy K., Xiong H., Chiu Y.L. (2010). Navitoclax, a targeted high-affinity inhibitor of BCL-2, in lymphoid malignancies: A phase 1 dose-escalation study of safety, pharmacokinetics, pharmacodynamics, and antitumour activity. Lancet Oncol..

[17] Roberts A.W., Seymour J.F., Brown J.R., Wierda W.G., Kipps T.J., Khaw S.L., Carney D.A., He S.Z., Huang D.C., Xiong H. (2012). Substantial susceptibility of chronic lymphocytic leukemia to BCL2 inhibition: Results of a phase I study of navitoclax in patients with relapsed or refractory disease. J. Clin. Oncol..

[18] Mason K.D., Carpinelli M.R., Fletcher J.I., Collinge J.E., Hilton A.A., Ellis S., Kelly P.N., Ekert P.G., Metcalf D., Roberts A.W. (2007). Programmed anuclear cell death delimits platelet life span. Cell.

[19] Zhang H., Nimmer P.M., Tahir S.K., Chen J., Fryer R.M., Hahn K.R., Iciek L.A., Morgan S.J., Nasarre M.C., Nelson R. (2007). Bcl-2 family proteins are essential for platelet survival. Cell Death Differ..

[20] Romanova E.I., Zubritskiy A.V., Lioznova A.V., Ogunleye A.J., Golotin V.A., Guts A.A., Lennartsson A., Demidov O.N., Medvedeva Y.A. (2022). RUNX1/CEBPA Mutation in Acute Myeloid Leukemia Promotes Hypermethylation and Indicates for Demethylation Therapy. Int. J. Mol. Sci..

[21] Koschade S.E., Stratmann J.A., Finkelmeier F., Wagner S., Chromik J., Steffen B., Serve H., Brandts C.H., Ballo O. (2022). Relapse surveillance of acute myeloid leukemia patients in first remission after consolidation chemotherapy: Diagnostic value of regular bone marrow aspirations. Ann. Hematol..

[22] Burnett A., Wetzler M., Lowenberg B. (2011). Therapeutic advances in acute myeloid leukemia. J. Clin. Oncol..

[23] Galluzzi L., Vitale I., Aaronson S.A., Abrams J.M., Adam D., Agostinis P., Alnemri E.S., Altucci L., Amelio I., Andrews D.W. (2018). Molecular mechanisms of cell death: Recommendations of the Nomenclature Committee on Cell Death 2018. Cell Death Differ..

[24] McBride A., Houtmann S., Wilde L., Vigil C., Eischen C.M., Kasner M., Palmisiano N. (2019). The Role of Inhibition of Apoptosis in Acute Leukemias and Myelodysplastic Syndrome. Front. Oncol..

[25] Seo W., Silwal P., Song I.C., Jo E.K. (2022). The dual role of autophagy in acute myeloid leukemia. J. Hematol. Oncol..

[26] Pattingre S., Tassa A., Qu X., Garuti R., Liang X.H., Mizushima N., Packer M., Schneider M.D., Levine B. (2005). Bcl-2 Antiapoptotic Proteins Inhibit Beclin 1-Dependent Autophagy. Cell.

[27] Altman J.K., Szilard A., Goussetis D.J., Sassano A., Colamonici M., Gounaris E., Frankfurt O., Giles F.J., Eklund E.A., Beauchamp E.M. (2014). Autophagy is a survival mechanism of acute myelogenous leukemia precursors during dual mTORC2/mTORC1 targeting. Clin. Cancer Res..

[28] Huang J., Sun M., Tao Y., Ren J., Peng M., Jing Y., Xiao Q., Yang J., Lin C., Lei L. (2023). Cytoplasmic Expression of TP53INP2 Modulated by Demethylase FTO and Mutant NPM1 Promotes Autophagy in Leukemia Cells. Int. J. Mol. Sci..

[29] Qian S., Wei Z., Yang W., Huang J., Yang Y., Wang J. (2022). The role of BCL-2 family proteins in regulating apoptosis and cancer therapy. Front. Oncol..

[30] Cheng T.C., Hong C., Akey I.V., Yuan S., Akey C.W. (2016). A near atomic structure of the active human apoptosome. eLife.

[31] Pfeffer C.M., Singh A.T.K. (2018). Apoptosis: A Target for Anticancer Therapy. Int. J. Mol. Sci..

[32] Molica M., Mazzone C., Niscola P., de Fabritiis P. (2021). TP53 Mutations in Acute Myeloid Leukemia: Still a Daunting Challenge?. Front. Oncol..

[33] Grob T., Al Hinai A.S.A., Sanders M.A., Kavelaars F.G., Rijken M., Gradowska P.L., Biemond B.J., Breems D.A., Maertens J., van Marwijk Kooy M. (2022). Molecular characterization of mutant TP53 acute myeloid leukemia and high-risk myelodysplastic syndrome. Blood.

[34] Grimwade D., Ivey A., Huntly B.J.P. (2016). Molecular landscape of acute myeloid leukemia in younger adults and its clinical relevance. Blood.

[35] Karakas T., Miething C.C., Maurer U., Weidmann E., Ackermann H., Hoelzer D., Bergmann L. (2002). The coexpression of the apoptosis-related genes bcl-2 and wt1 in predicting survival in adult acute myeloid leukemia. Leukemia.

[36] Handschuh L., Wojciechowski P., Kazmierczak M., Lewandowski K. (2021). Transcript-Level Dysregulation of BCL2 Family Genes in Acute Myeloblastic Leukemia. Cancers.

[37] Martínez-Laperche C., Kwon M., Franco-Villegas A.C., Chillón M.C., Castro N., Anguita E., Dolz S., Rodríguez-Medina C., Hermosín L., Bellón J.M. (2018). Wilms Tumor 1 gene expression levels improve risk stratification in AML patients. Results of a multicentre study within the Spanish Group for Molecular Biology in Haematology. Br. J. Haematol..

[38] Kwon M., Martínez-Laperche C., Infante M., Carretero F., Balsalobre P., Serrano D., Gayoso J., Pérez-Corral A., Anguita J., Díez-Martín J.L. (2012). Evaluation of minimal residual disease by real-time quantitative PCR of Wilms’ tumor 1 expression in patients with acute myelogenous leukemia after allogeneic stem cell transplantation: Correlation with flow cytometry and chimerism. Biol. Blood Marrow Transplant..

[39] Giudice V., Gorrese M., Vitolo R., Bertolini A., Marcucci R., Serio B., Guariglia R., Ferrara I., Pepe R., D’Alto F. (2021). *WT1* Expression Levels Combined with Flow Cytometry Blast Counts for Risk Stratification of Acute Myeloid Leukemia and Myelodysplastic Syndromes. Biomedicines.

[40] Ng S.W., Mitchell A., Kennedy J.A., Chen W.C., McLeod J., Ibrahimova N., Arruda A., Popescu A., Gupta V., Schimmer A.D. (2016). A 17-gene stemness score for rapid determination of risk in acute leukaemia. Nature.

[41] Herold T., Jurinovic V., Batcha A.M.N., Bamopoulos S.A., Rothenberg-Thurley M., Ksienzyk B., Hartmann L., Greif P.A., Phillippou-Massier J., Krebs S. (2018). A 29-gene and cytogenetic score for the prediction of resistance to induction treatment in acute myeloid leukemia. Haematologica.

[42] Findley H.W., Gu L., Yeager A.M., Zhou M. (1997). Expression and regulation of Bcl-2, Bcl-xl, and Bax correlate with p53 status and sensitivity to apoptosis in childhood acute lymphoblastic leukemia. Blood.

[43] Sillar J.R., Enjeti A.K. (2019). Targeting Apoptotic Pathways in Acute Myeloid Leukaemia. Cancers.

[44] Montero J., Letai A. (2018). Why do BCL-2 inhibitors work and where should we use them in the clinic?. Cell Death Differ..

[45] Kim R., Emi M., Tanabe K., Toge T. (2004). Therapeutic potential of antisense Bcl-2 as a chemosensitizer for cancer therapy. Cancer.

[46] Nguyen M., Marcellus R.C., Roulston A., Watson M., Serfass L., Murthy Madiraju S.R., Goulet D., Viallet J., Bélec L., Billot X. (2007). Small molecule obatoclax (GX15-070) antagonizes MCL-1 and overcomes MCL-1-mediated resistance to apoptosis. Proc. Natl. Acad. Sci. USA.

[47] Konopleva M., Watt J., Contractor R., Tsao T., Harris D., Estrov Z., Bornmann W., Kantarjian H., Viallet J., Samudio I. (2008). Mechanisms of antileukemic activity of the novel Bcl-2 homology domain-3 mimetic GX15-070 (obatoclax). Cancer Res..

[48] van Delft M.F., Wei A.H., Mason K.D., Vandenberg C.J., Chen L., Czabotar P.E., Willis S.N., Scott C.L., Day C.L., Cory S. (2006). The BH3 mimetic ABT-737 targets selective Bcl-2 proteins and efficiently induces apoptosis via Bak/Bax if Mcl-1 is neutralized. Cancer Cell.

[49] Kuusanmäki H., Dufva O., Vähä-Koskela M., Leppä A.M., Huuhtanen J., Vänttinen I., Nygren P., Klievink J., Bouhlal J., Pölönen P. (2023). Erythroid/megakaryocytic differentiation confers BCL-XL dependency and venetoclax resistance in acute myeloid leukemia. Blood.

[50] Haemmerle M., Stone R.L., Menter D.G., Afshar-Kharghan V., Sood A.K. (2018). The Platelet Lifeline to Cancer: Challenges and Opportunities. Cancer Cell.

[51] Park C.H., Yun J.W. (2022). Investigation of Biomarkers Associated with Low Platelet Counts in Normal Karyotype Acute Myeloid Leukemia. Int. J. Mol. Sci..

[52] Souers A.J., Leverson J.D., Boghaert E.R., Ackler S.L., Catron N.D., Chen J., Dayton B.D., Ding H., Enschede S.H., Fairbrother W.J. (2013). ABT-199, a potent and selective BCL-2 inhibitor, achieves antitumor activity while sparing platelets. Nat. Med..

[53] Skopek R., Palusińska M., Kaczor-Keller K., Pingwara R., Papierniak-Wyglądała A., Schenk T., Lewicki S., Zelent A., Szymański Ł. (2023). Choosing the Right Cell Line for Acute Myeloid Leukemia (AML) Research. Int. J. Mol. Sci..

[54] Konopleva M., Pollyea D.A., Potluri J., Chyla B., Hogdal L., Busman T., McKeegan E., Salem A.H., Zhu M., Ricker J.L. (2016). Efficacy and Biological Correlates of Response in a Phase II Study of Venetoclax Monotherapy in Patients with Acute Myelogenous Leukemia. Cancer Discov..

[55] Tsao T., Shi Y., Kornblau S., Lu H., Konoplev S., Antony A., Ruvolo V., Qiu Y.H., Zhang N., Coombes K.R. (2012). Concomitant inhibition of DNA methyltransferase and BCL-2 protein function synergistically induce mitochondrial apoptosis in acute myelogenous leukemia cells. Ann. Hematol..

[56] Wolach O., Frisch A., Shargian L., Yeshurun M., Apel A., Vainstein V., Moshe Y., Shimony S., Amit O., Bar-On Y. (2022). Venetoclax in combination with FLAG-IDA-based protocol for patients with acute myeloid leukemia: A real-world analysis. Ann. Hematol..

[57] DiNardo C.D., Jonas B.A., Pullarkat V., Thirman M.J., Garcia J.S., Wei A.H., Konopleva M., Döhner H., Letai A., Fenaux P. (2020). Azacitidine and Venetoclax in Previously Untreated Acute Myeloid Leukemia. N. Engl. J. Med..

[58] Chonghaile T.N., Roderick J.E., Glenfield C., Ryan J., Sallan S.E., Silverman L.B., Loh M.L., Hunger S.P., Wood B., DeAngelo D.J. (2014). Maturation stage of T-cell acute lymphoblastic leukemia determines BCL-2 versus BCL-XL dependence and sensitivity to ABT-199. Cancer Discov..

[59] Pullarkat V.A., Lacayo N.J., Jabbour E., Rubnitz J.E., Bajel A., Laetsch T.W., Leonard J., Colace S.I., Khaw S.L., Fleming S.A. (2021). Venetoclax and Navitoclax in Combination with Chemotherapy in Patients with Relapsed or Refractory Acute Lymphoblastic Leukemia and Lymphoblastic Lymphoma. Cancer Discov..

[60] Khaw S.L., Suryani S., Evans K., Richmond J., Robbins A., Kurmasheva R.T., Billups C.A., Erickson S.W., Guo Y., Houghton P.J. (2016). Venetoclax responses of pediatric ALL xenografts reveal sensitivity of MLL-rearranged leukemia. Blood.

[61] Aumann S., Shaulov A., Haran A., Gross Even-Zohar N., Vainstein V., Nachmias B. (2022). The Emerging Role of Venetoclax-Based Treatments in Acute Lymphoblastic Leukemia. Int. J. Mol. Sci..

[62] Pan R., Hogdal L.J., Benito J.M., Bucci D., Han L., Borthakur G., Cortes J., DeAngelo D.J., Debose L., Mu H. (2014). Selective BCL-2 inhibition by ABT-199 causes on-target cell death in acute myeloid leukemia. Cancer Discov..

[63] Konopleva M., Contractor R., Tsao T., Samudio I., Ruvolo P.P., Kitada S., Deng X., Zhai D., Shi Y.X., Sneed T. (2006). Mechanisms of apoptosis sensitivity and resistance to the BH3 mimetic ABT-737 in acute myeloid leukemia. Cancer Cell.

[64] Del Gaizo Moore V., Brown J.R., Certo M., Love T.M., Novina C.D., Letai A. (2007). Chronic lymphocytic leukemia requires BCL2 to sequester prodeath BIM, explaining sensitivity to BCL2 antagonist ABT-737. J. Clin. Investig..

[65] Zhang W., Konopleva M., Burks J.K., Dywer K.C., Schober W.D., Yang J.Y., McQueen T.J., Hung M.C., Andreeff M. (2010). Blockade of mitogen-activated protein kinase/extracellular signal-regulated kinase kinase and murine double minute synergistically induces Apoptosis in acute myeloid leukemia via BH3-only proteins Puma and Bim. Cancer Res..

[66] Wei A.H., Montesinos P., Ivanov V., DiNardo C.D., Novak J., Laribi K., Kim I., Stevens D.A., Fiedler W., Pagoni M. (2020). Venetoclax plus LDAC for newly diagnosed AML ineligible for intensive chemotherapy: A phase 3 randomized placebo-controlled trial. Blood.

[67] Wang H., Mao L., Yang M., Qian P., Lu H., Tong H., Xie W., Zhou D., Huang X., Wang Y. (2022). Venetoclax plus 3 + 7 daunorubicin and cytarabine chemotherapy as first-line treatment for adults with acute myeloid leukaemia: A multicentre, single-arm, phase 2 trial. Lancet Haematol..

[68] Kadia T.M., Reville P.K., Borthakur G., Yilmaz M., Kornblau S., Alvarado Y., Dinardo C.D., Daver N., Jain N., Pemmaraju N. (2021). Venetoclax plus intensive chemotherapy with cladribine, idarubicin, and cytarabine in patients with newly diagnosed acute myeloid leukaemia or high-risk myelodysplastic syndrome: A cohort from a single-centre, single-arm, phase 2 trial. Lancet Haematol..

[69] Piciocchi A., Marconi G., Cipriani M., Messina M., Guolo F., Minetto P., Paoloni F., Cotugno F., Di Donato L., Simonetti G. (2023). P523: The comparison of vflai, flai and 3 + 7 regimens by multilevel propensity score weighting highlights the benefit of the addition of venetoclax in no low-risk aml treated in gimema trials and real world. HemaSphere.

[70] Pollyea D.A., DiNardo C.D., Arellano M.L., Pigneux A., Fiedler W., Konopleva M., Rizzieri D.A., Smith B.D., Shinagawa A., Lemoli R.M. (2022). Impact of Venetoclax and Azacitidine in Treatment-Naïve Patients with Acute Myeloid Leukemia and IDH1/2 Mutations. Clin. Cancer Res..

[71] Lachowiez C.A., Loghavi S., Kadia T.M., Daver N., Borthakur G., Pemmaraju N., Naqvi K., Alvarado Y., Yilmaz M., Short N. (2020). Outcomes of older patients with NPM1-mutated AML: Current treatments and the promise of venetoclax-based regimens. Blood Adv..

[72] Bataller A., Loghavi S., Gerstein Y., Bazinet A., Sasaki K., Chien K.S., Hammond D., Montalban-Bravo G., Borthakur G., Short N. (2023). Characteristics and clinical outcomes of patients with myeloid malignancies and DDX41 variants. Am. J. Hematol..

[73] Nanaa A., He R., Foran J.M., Badar T., Gangat N., Pardanani A., Hogan W.J., Litzow M.R., Patnaik M., Al-Kali A. (2023). Venetoclax plus hypomethylating agents in DDX41-mutated acute myeloid leukaemia and myelodysplastic syndrome: Mayo Clinic series on 12 patients. Br. J. Haematol..

[74] Gangat N., McCullough K., Johnson I., Al-Kali A., Begna K.H., Patnaik M.M., Litzow M.R., Hogan W., Shah M., Alkhateeb H. (2022). Real-world experience with venetoclax and hypomethylating agents in myelodysplastic syndromes with excess blasts. Am. J. Hematol..

[75] DiNardo C.D., Tiong I.S., Quaglieri A., MacRaild S., Loghavi S., Brown F.C., Thijssen R., Pomilio G., Ivey A., Salmon J.M. (2020). Molecular patterns of response and treatment failure after frontline venetoclax combinations in older patients with AML. Blood.

[76] Ong F., Kim K., Konopleva M.Y. (2022). Venetoclax resistance: Mechanistic insights and future strategies. Cancer Drug Resist..

[77] Arslan S., Zhang J., Dhakal P., Moran J., Naidoo N., Lombardi J., Pullarkat V., Stein A.S., Marcucci G., Yaghmour G. (2021). Outcomes of therapy with venetoclax combined with a hypomethylating agent in favorable-risk acute myeloid leukemia. Am. J. Hematol..

[78] Zhang K., Zhang X., Xu Y., Xue S., Qiu H., Tang X., Han Y., Chen S., Sun A., Zhang Y. (2023). Efficacy of venetoclax combined with hypomethylating agents in young, and unfit patients with newly diagnosed core binding factor acute myeloid leukemia. Blood Cancer J..

[79] Chen S., Xie J., Yang X., Shen H., Cen J., Yao L., Hu X., Wu Q., Zhang J., Qiu H. (2021). Venetoclax Plus Decitabine for Young Adults with Newly Diagnosed ELN Adverse-Risk Acute Myeloid Leukemia: Interim Analysis of a Prospective, Multicenter, Single-Arm, Phase 2 Trial. Blood.

[80] Suo X., Zheng F., Wang D., Zhao L., Liu J., Li L., Zhang Z., Zhang C., Li Y., Yang S. (2023). Venetoclax combined with daunorubicin and cytarabine (2 + 6) as induction treatment in adults with newly diagnosed acute myeloid leukemia: A phase 2, multicenter, single-arm trial. Exp. Hematol. Oncol..

[81] Piciocchi A., Messina M., Cipriani M., Paoloni F., Simonetti G., Palmieri R., Marconi G., Buccisano F., Fazi P., Vignetti M. (2022). The Addition of Venetoclax to Induction Chemotherapy in No Low-Risk AML Patients: A Propensity Score-Matched Analysis of the Gimema AML1718 and AML1310 Trials. Blood.

[82] Tausch E., Close W., Dolnik A., Bloehdorn J., Chyla B., Bullinger L., Döhner H., Mertens D., Stilgenbauer S. (2019). Venetoclax resistance and acquired BCL2 mutations in chronic lymphocytic leukemia. Haematologica.

[83] Blombery P., Lew T.E., Dengler M.A., Thompson E.R., Lin V.S., Chen X., Nguyen T., Panigrahi A., Handunnetti S.M., Carney D.A. (2022). Clonal hematopoiesis, myeloid disorders and BAX-mutated myelopoiesis in patients receiving venetoclax for CLL. Blood.

[84] Roberts A.W., Ma S., Kipps T.J., Coutre S.E., Davids M.S., Eichhorst B., Hallek M., Byrd J.C., Humphrey K., Zhou L. (2019). Efficacy of venetoclax in relapsed chronic lymphocytic leukemia is influenced by disease and response variables. Blood.

[85] Herling C.D., Abedpour N., Weiss J., Schmitt A., Jachimowicz R.D., Merkel O., Cartolano M., Oberbeck S., Mayer P., Berg V. (2018). Clonal dynamics towards the development of venetoclax resistance in chronic lymphocytic leukemia. Nat. Commun..

[86] Khalsa J.K., Cha J., Utro F., Naeem A., Murali I., Kuang Y., Vasquez K., Li L., Tyekucheva S., Fernandes S.M. (2023). Genetic events associated with venetoclax resistance in CLL identified by whole-exome sequencing of patient samples. Blood.

[87] Tiong I.S., Dillon R., Ivey A., Teh T.C., Nguyen P., Cummings N., Taussig D.C., Latif A.L., Potter N.E., Runglall M. (2021). Venetoclax induces rapid elimination of NPM1 mutant measurable residual disease in combination with low-intensity chemotherapy in acute myeloid leukaemia. Br. J. Haematol..

[88] Sartor C., Brunetti L., Audisio E., Cignetti A., Zannoni L., Cristiano G., Nanni J., Ciruolo R., Zingarelli F., Ottaviani E. (2023). A venetoclax and azacitidine bridge-to-transplant strategy for NPM1-mutated acute myeloid leukaemia in molecular failure. Br. J. Haematol..

[89] Chan S.M., Thomas D., Corces-Zimmerman M.R., Xavy S., Rastogi S., Hong W.J., Zhao F., Medeiros B.C., Tyvoll D.A., Majeti R. (2015). Isocitrate dehydrogenase 1 and 2 mutations induce BCL-2 dependence in acute myeloid leukemia. Nat. Med..

[90] Leverson J.D., Phillips D.C., Mitten M.J., Boghaert E.R., Diaz D., Tahir S.K., Belmont L.D., Nimmer P., Xiao Y., Ma X.M. (2015). Exploiting selective BCL-2 family inhibitors to dissect cell survival dependencies and define improved strategies for cancer therapy. Sci. Transl. Med..

[91] Chyla B., Daver N., Doyle K., McKeegan E., Huang X., Ruvolo V., Wang Z., Chen K., Souers A., Leverson J. (2018). Genetic biomarkers of sensitivity and resistance to venetoclax monotherapy in patients with relapsed acute myeloid leukemia. Am. J. Hematol..

[92] Lachowiez C.A., Reville P.K., Kantarjian H., Jabbour E., Borthakur G., Daver N., Issa G., Furudate K., Tanaka T., Pierce S. (2022). Contemporary outcome in IDH-mutated acute myeloid leukemia: The impact of co-occuring NPM1 mutations and venetoclax-based treatment. Am. J. Hematol..

[93] Stuani L., Sabatier M., Saland E., Cognet G., Poupin N., Bosc C., Castelli F.A., Gales L., Turtoi E., Montersino C. (2021). Mitochondrial metabolism supports resistance to IDH mutant inhibitors in acute myeloid leukemia. J. Exp. Med..

[94] Rahmani N.E., Ramachandra N., Sahu S., Gitego N., Lopez A., Pradhan K., Bhagat T.D., Gordon-Mitchell S., Pena B.R., Kazemi M. (2021). ASXL1 mutations are associated with distinct epigenomic alterations that lead to sensitivity to venetoclax and azacytidine. Blood Cancer J..

[95] Lachowiez C.A., Loghavi S., Furudate K., Montalban-Bravo G., Maiti A., Kadia T., Daver N., Borthakur G., Pemmaraju N., Sasaki K. (2021). Impact of splicing mutations in acute myeloid leukemia treated with hypomethylating agents combined with venetoclax. Blood Adv..

[96] Senapati J., Urrutia S., Loghavi S., Short N.J., Issa G.C., Maiti A., Abbas H.A., Daver N.G., Pemmaraju N., Pierce S. (2023). Venetoclax abrogates the prognostic impact of splicing factor gene mutations in newly diagnosed acute myeloid leukemia. Blood.

[97] Rodríguez-Afonso J., Reyes-González-Casanova P., Álvarez M.A., Bilbao C., Stuckey R., López J.F., Medina C., Ortega Y., Cionfrini A., Sosa S. (2023). PB1893: Clonal evolution with bcl-2 amplification during venetoclax treatment. HemaSphere.

[98] Xu Y., Ye H. (2022). Progress in understanding the mechanisms of resistance to BCL-2 inhibitors. Exp. Hematol. Oncol..

[99] Lee J.B., Khan D.H., Hurren R., Xu M., Na Y., Kang H., Mirali S., Wang X., Gronda M., Jitkova Y. (2021). Venetoclax enhances T cell-mediated antileukemic activity by increasing ROS production. Blood.

[100] Vo T.T., Ryan J., Carrasco R., Neuberg D., Rossi D.J., Stone R.M., Deangelo D.J., Frattini M.G., Letai A. (2012). Relative mitochondrial priming of myeloblasts and normal HSCs determines chemotherapeutic success in AML. Cell.

[101] Gjertsen B.T. (2023). How to discover the exceptional venetoclax responders in AML/MDS?. Br. J. Haematol..

